# Fly Ash with Ammonia: Properties and Emission of Ammonia from Cement Composites

**DOI:** 10.3390/ma14040707

**Published:** 2021-02-03

**Authors:** Filip Chyliński, Anna Goljan, Agnieszka Michalik

**Affiliations:** Building Research Institute, Filtrowa 1, 00-611 Warsaw, Poland; a.goljan@itb.pl (A.G.); a.michalik@itb.pl (A.M.)

**Keywords:** fly ash, ammonia, denitrification process, ammonia slip, ammonia emission, concrete

## Abstract

The article presents the results of tests performed on fly ash with a high content of ammonium (up to 400 ppm) from the NOx reduction process. The main properties of fly ash were tested according to EN 450-1 and the results were compared with fly ash without ammonium. The comparison showed that fly ash with high concentration of ammonium suits the requirements of the European standard. Although the requirements do not limit the ammonium content, using such material as an additive for cement composites causes the emission of gaseous ammonium during mixing and from the final product. For this reason, the emission of ammonium from mortars containing fly ash were tested. The results have shown that using high ammonium fly ash might pollute indoor air and affect the health of users.

## 1. Introduction

Fly ash is a widely used by-product, no longer considered just a waste but a rather a valuable pozzolanic material added to cement composites to improve their properties [1,2,3,4,5,6,7]. It is used in cement and concrete manufacturing after fulfilling the requirements of various standards such as EN 197-1, EN 450-1 and ASTM C618 [8,9,10,11]. The world production of siliceous fly ash in 2016 was about 550 million tonnes [12].

Since 2010, when the EU members signed Directive 2010/75/EU on industrial emissions (integrated pollution prevention and control), the so-called IED Directive [13], the levels of emission of hazardous constituents of flue gases from power plants, including NOx, have been reduced. The level of NOx emissions has been reduced to 300 to 150 mg/Nm^3^ depending on the total rated thermal input of the combustion power plant. In order to meet the new requirements, the coal burning process had to be modified, in most cases by adding an installation for reducing nitrogen oxides. Two of the most popular installations are Selective Non-Catalytic Reduction (SNCR) and Selective Catalytic Reduction (SCR) [14,15,16]. Both installations use a reducing agent, which is injected directly to the combustion chamber (in SNCR) or into the flue gas before entering the catalytic zone. The reducing agent is a source of ammonium, which reduces the concentration of NOx and generates nitrogen. Due to dynamic processes, however, not all ammonium particles react, with some remaining in the flue gas, which is called “ammonia slip”. Part of the ammonium adsorbs on the surface of the spherical particles of fly ash and part reacts with other constituents of flue gas, forming ammonium sulphates, carbonates or chlorides. The ammonium content in fly ash depends on many factors [17,18] among which the main one is the ammonia slip level. Mazur et al. [19] have estimated that an ammonia overdose of 2 ppm might increase the ammonia content in fly ash by about 100 ppm. Fly ashes from power plants might even contain up to 2500 ppm of ammonium [20]. Due to its unpleasant odour and its harmful effects, good practice in the market dictates that fly ash should contain less than 100 ppm of ammonia, and in some countries like Germany, as little as 50 ppm [17,19,21].

The EN 450-1 and ASTM C618 standards [9,11], which contain the requirements for the properties of siliceous fly ash suitable for use as an additive for concrete, do not limit the ammonium content in fly ash. Similarly, the EN 197-1 standard [8] containing the requirements for fly ash used as part of cement does not limit the NH_3_ content.

Due to the lack of requirements for ammonium content in fly ash, the requirements for indoor air and volatile organic compounds (VOC) emissions from building materials could be adapted.

According to the US Department of Health and Human Services [22,23], long-term exposure to air with an NH_3_ concentration above 1 ppm can cause irritation of the eyes, nose and throat. The Occupational Safety and Health Administration (OSHA) limits exposure to air with 35 ppm of ammonia to no longer than 15 min and to air with 50 ppm to no longer than 5 min [24]. The British Health and Safety Executive limits the ammonia concentration in air at the workplace to 18 mg/m^3^ for no longer than 8 h [25]. At a level of 5 ppm or more, the odour of ammonia can be detected [26]. Only a handful of countries regulate the limits of ammonia content in indoor air in the workplace. A Finnish voluntary declaration [27] limits the emission of ammonia from building products into the indoor air to 0.03 or 0.06 mg/m^2^ h, depending on the type of room. A Polish national law project [28] will limit the ammonium content in indoor air to 300 or 100 µg/m^3^ depending on exposure—under or above 4 h a day. According to the European Registration, Evaluation, Authorisation and Restriction of Chemicals (REACH) directive [29], building materials containing inorganic ammonium salts are not to be marketed when the emission of ammonia (in special conditions) is higher than 3 ppm.

The emission of ammonia from cement composites was investigated by Hongseok et al. [22] who observed a rise of ammonia content in air from mortar with aggregate containing organic matter. Bai et. al. [30] investigated the emissions of ammonia from concrete containing urea-based antifreeze admixtures showing that this agent might be also a source of ammonia emissions. Shou et al. [31] measured the emissions of ammonia from concrete containing fly ash from the denitrification process. The level of ammonia content in air ranged from 65 to 3200 mg NH_4_^+^/kg fly ash where 80% of the emissions occurred during the first 8 h. The gas release was observed to last up to three weeks and after that the emissions reached a negligible level. Bentur and Larianovsky [32] measured the emission of ammonia from concrete mixes with fly ash and ammonium salts, starting from the first contact of binder with water. They registered the highest emission peak (up to 40 ppm) during the first 2 h. The maximum content of ammonia in the fly ash was 180 ppm.

The aim of this article was to answer the question of whether siliceous fly ash containing up to 400 ppm of ammonia might still be a useful and safe material for concrete production and service or whether it needs to be treated as a waste and lanfilled. In order to answer this question, a comparison of the properties of fly ash with high content of ammonia to fly ash without ammonia and the requirements stated in EN 450-1 [9] was made. The emission of gaseous ammonia from mortars with fly ash was also tested to check whether this building material would fulfil the requirements for the emission of VOCs and indoor air quality.

The article presents the results of tests chosen by previous investigations [18] that showed that these properties might be significant for fly ash containing ammonia, and also other tests found valuable in this area. The following tests were performed:

- loss on ignition
- fineness and water requirement
- properties of fresh mortar
- setting time
- compressive and bending strength
- pozzolanic activity
- emission of ammonia from mortars

## 2. Materials and Methods

### 2.1. Fly Ash and Cement

Tests were performed using siliceous fly ash from a Polish power plant equipped with an SNCR denitrification installation (samples A-E). The reference sample (REF) was from another Polish power plant without an installation for the denitrification of flue gases. Fly ash was certified according to the EN 450-1 standard [9] as fly ash Category A for concrete. Table 1 presents the ammonia content of the tested fly ash. The ammonium content in the fly ash at the day of arrival from the power plant was determined by leaching it into water (water: solid ratio—10:1) and determining the ammonium concentration in the water according to the EN 12457-4 standard [33].

In order to prepare grouts and mortars, Portland cement CEM I 42,5R was used. The cement was certified according to the EN 197-1 standard [8]. Table 2 and Table 3 present the main characteristics and composition of the cement used for the tests.

### 2.2. Loss on Ignition, Fineness and Water Requirement

For each sample of fly ash, the loss on ignition was tested according to EN 196-2 and EN 450-1 [9,34]. The fineness of fly ash was tested according to EN 451-2 [35]. Water requirement was tested according to EN 450-1 Annex B [9]. The tests were repeated three times for each sample and the average value was calculated.

### 2.3. Properties of Fresh Mortar

**Figure 1:**
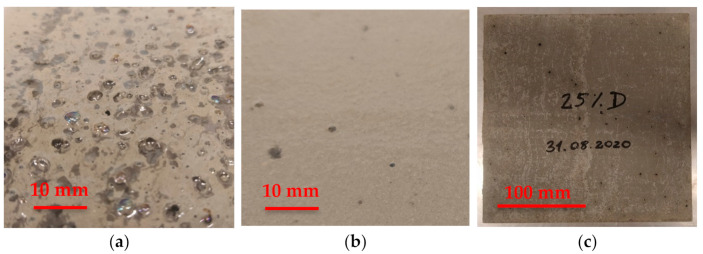
Mortar emitting gaseous ammonia, (**a**) after compaction, (**b**) a few seconds after floating the surface, (**c**) hardened mortar with visible holes (black dots).

Due to the observation that bubbles of gaseous ammonia appear on the surface of fresh mortar (Figure 1), the properties of the fresh mortar were tested to check if gaseous ammonia affects the results of tests of air content, density and consistency. Air content, density and consistency were tested according to EN 1015-7, EN 1015-6 and EN 1015-3 respectively [36,37,38,39].

### 2.4. Setting Time

Tests on the influence of fly ash on the setting time of cement grouts were performed according to EN 196-3 and EN-450-1 [9,40]. The compositions of the tested grouts are given in Table 4.

### 2.5. Compressive and Bending Strength

Mortars with the compositions from Table 5 were mixed and placed into prismatic moulds with dimensions of 160 mm × 40 mm × 40 mm according to EN 196-1 [41]. The following day, after demoulding, the samples were put into water at a temperature of 20 ± 2 °C and cured until the day of the test. Compressive and flexural strength were tested according to EN 196-1 [41] after 28, 90 and 180 days. The addition of fly ash was 25% and 50% of the binder mass. The amount of 25% of fly ash is typically used in the production of concrete and mentioned in the EN 206 standard [42] as the maximum amount to put in the place of CEM II/A Portland cement (higher amounts of fly ash might be used but cannot be treated as binders but rather as fillers). The amount of 50% of ash was used to check whether this significantly exceeded amount of fly ash with a high content of ammonia would affect the properties of the grout or mortar. Samples A, C and E of fly ash were chosen as the representative one for the low, medium and high concentration of ammonia to observe if there is an influence of ammonia for the tested properties of mortars.

### 2.6. Pozzolanic Activity

The pozzolanic activity index was calculated by comparing the compressive strength values of mortars with fly ash to those of mortars without fly ash. According to EN 450-1 [9], a comparison should be made with samples containing 25% of fly ash (in the place of cement) and the reference sample (without fly ash).

### 2.7. Emission of Ammonia from Mortars

**Figure 2:**
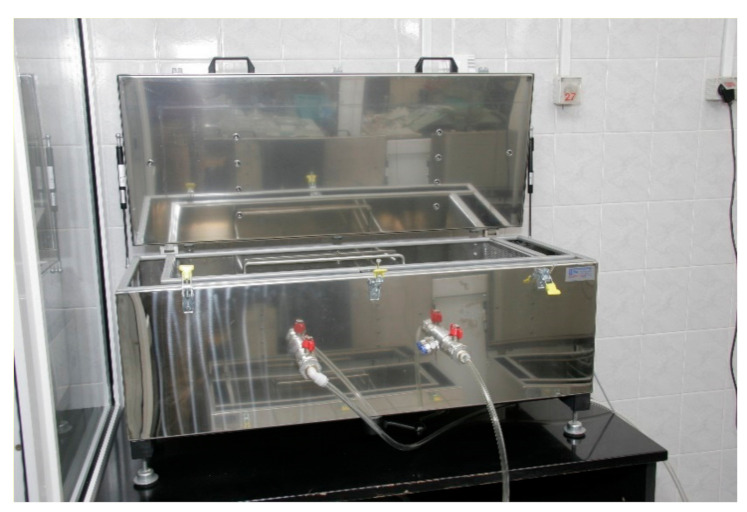
Testing chamber.

Tests on the emission of ammonia from mortars were performed to check how the ammonium from the fly ash affects the indoor air in buildings and whether they are safe to use. The tests were carried out in a testing chamber (Figure 2). The testing chamber conformed to the requirements of EN 16516 [43]. According to EN 16516 [43], the surface area of the tested sample should be chosen depending on the scenario for the model room. The model room has the following dimensions:

- height 2.5 m
- floor (and ceiling) 3 m × 4 m
- one door with dimensions 0.8 m × 2 m
- one window with a surface of 2 m^2^

The sum of the area of the walls (without the doors and window) is 31.4 m^2^ and the volume is 30 m^3^. The loading factor in the model room (or the testing chamber) is calculated by the proportion of the area taken by the product (e.g., walls, ceiling) to the volume of the model room according to Equation (1):

(1) $$L=\frac{P}{V}$$

where L—loading factor (m^2^/m^3^); P—area of intended use of the product (m^2^); V—volume of the testing chamber (m^3^).

Depending on the area of intended use of the product, the following loading factor values might be used:

- 1.0 m^2^/m^3^ for the wall emission
- 0.4 m^2^/m^3^ for the floor or ceiling emissions
- 1.8 m^2^/m^3^ for the emission from walls, floor and ceiling

For the emission tests, samples D and E were chosen as the ones with the highest concentration of ammonia to observe if there is an emission of ammonia from mortars. Mortars with 15, 25 and 50% b.m. (binder mass) of fly ash were prepared for the test. The compositions of the tested mortars are given in Table 6.

The surface area of the tested mortar samples was 0.1 m^2^ and the volume of the chamber was 0.1 m^3^. The saturation index was 1 m^2^/m^3^, which is suitable for the scenario of testing emissions from the walls in the reference room. The emission tests were carried out in the sealed steel laboratory chamber shown in Figure 2, which fulfils the requirements of EN 16516 [43].

**Figure 3:**
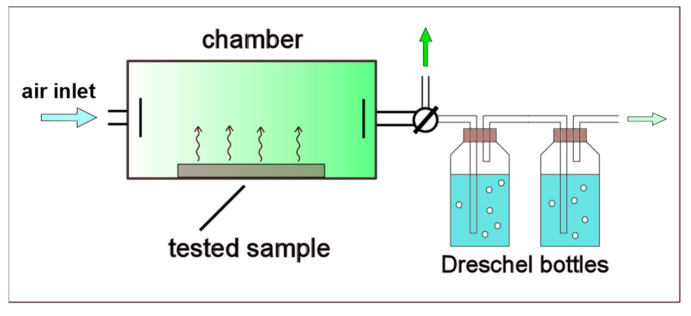
Scheme of the testing installation.

The scheme of the testing installation is shown in Figure 3.

**Figure 4:**
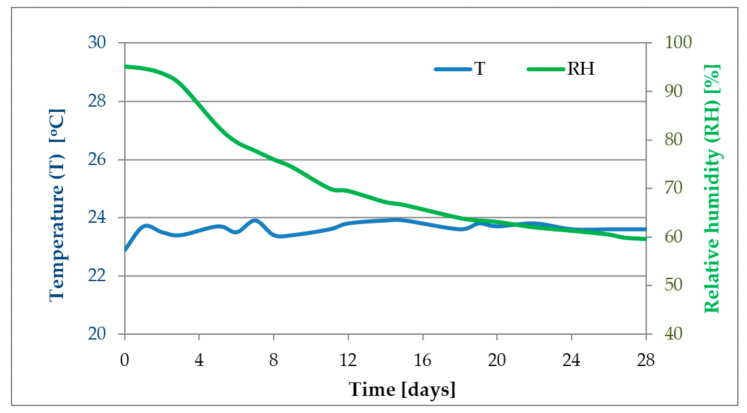
Temperature and relevant humidity during the test for ammonia emissions.

Airflow in the chamber was 0.05 m^3^/h (half of the volume of the chamber per hour). The temperature and humidity were measured during the test and the results are shown in Figure 4.

In order to sample ammonia, the chamber air outlet channel was connected in parallel with two Dreschel bottles filled with 30 mL of 0.01 M H_2_SO_4_ solution, where the gaseous NH_3_ was absorbed. The sample was collected at a rate of 500 mL/min for 2 h using a GilAir Plus pump (Gilian, St. Petersburg, FL, USA)

The concentration of ammonia in the solution was analysed using a Metrohm 930 Compact IC Flex ion chromatograph equipped with a conductometry detector (Metrohm AG, Herisau, Switzerland). The chromatograph was equipped with a precolumn Metrosep C4 Guard/4.0 (length 5 mm and diameter 4 mm) and a Metrosep C6 column (length 150 mm and diameter 4 mm) filled with silica gel with carboxylic groups (particle size 5 μm). The eluent used for analysis was a 1.7 mmol/L nitric acid and 1.7 mmol/L dipicolinic acid. The eluent flow during the analysis was 0.9 mL/min, the temperature was 25 °C and the pressure of the eluent was 6.87 MPa. The volume of injection was 20 µL and the duration of analysis was 25 min. The concentration of ammonia in each of the Dreschel bottles was summarised and the concentration in the chamber air was calculated. The emission tests were carried for 28 days.

## 3. Results and Discussion

### 3.1. Loss on Ignition, Fineness and Water Requirement

Table 7 presents the average values of loss on ignition, fineness and water demand of fly ashes.

The results of tests in Table 7 show that the REF fly ash exhibits the lowest loss on ignition, which might be related with the modifications of the combustion chamber in the power plant aimed at lowering the amount of generated NO*x*. This is known to have an effect on different conditions during the burning of coal and lower temperatures. No correlation was found between the ammonium content in fly ash and its loss on ignition.

Fly ashes with lower fineness had the highest concentration of ammonia, which suggests that fly ashes with smaller grains and in fact higher surface might absorb higher amounts of ammonia. The correlation between those parameters was quite low with a coefficient of determination R^2^ = 0.76, which shows that if fineness determines the ammonia content it is not the only parameter.

The results of water demand of fly ashes show no influence of ammonia content on this parameter. The results of the tests of loss on ignition, fineness and water demand thus show that siliceous fly ash containing up to 400 ppm of ammonia might still be a useful material for concrete production.

### 3.2. Properties of Fresh Mortar

Table 8 presents the results of tests on the properties of fresh mortar.

The density of fresh mortars is related with their air content and the amount of fly ash used in the place of cement due to their different density. The air content in fresh mortar might be affected by the generation of gaseous ammonia, which raises the “air” content, and also by the amount of unburned coal particles, which decreases air content [44,45]. Fly ash with the highest concentration of ammonia from those tested (E) and in the highest amount (50% E) increases the amount of air entered into the mortar, which in effect gives the lowest density of mortar. The results of the tests did not show any straight correlation between the ammonia content in fly ash and the density or air content.

The consistency of fresh mortar is related to factors such as the shape of particles, air content and the fineness of fly ash. There is no straight correlation between the content of ammonia and the consistency of fresh mortar. The lowest value of consistency was shown by the “100% CEM I” sample with no fly ash, while the “50% Ref” sample with the highest amount of fly ash and without ammonia had the highest consistency. These results can be explained by the properties of fly ash, which acts as a plasticising agent [46].

Results of tests on the properties of fresh mortar did not showed any potential risk of using fly ash containing up to 400 ppm of ammonia as an addition to concrete.

### 3.3. Setting Time

Table 9 presents the results of setting time tests of grouts with compositions described in Table 4. The addition of fly ash to grout increases its setting time, which is as per EN 450-1 [9]. In analysing the results of the performed tests, however, it has been observed that fly ash containing ammonia increases the setting time even more, which is also confirmed in the literature [47].

**Figure 5:**
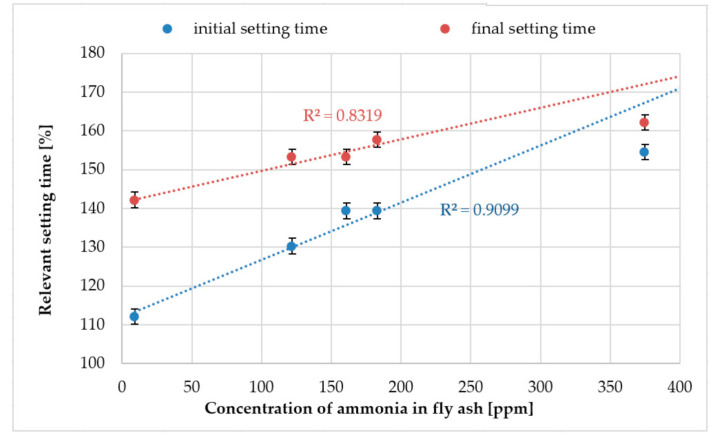
Correlation between ammonia content and relevant setting time.

Figure 5 shows the plots and correlation between the ammonia concentration in fly ash and the initial and final setting times of grouts. The correlation between those parameters sits quite well with the coefficient of determination R^2^ = 0.91 and 0.83 for the initial and final setting times respectively. According to the EN 450-1 standard [9], the relevant initial setting time for fly ash used for the concrete should not be higher than 200%. Tested fly ash containing up to 400 ppm of ammonia fulfils this requirement, however extrapolating the data from Figure 5, it can be calculated that fly ash containing more than 600 ppm of ammonia will not fulfil this requirement and cannot be used as an additive for concrete.

### 3.4. Compressive Strength, Bending Strength and Pozzolanic Activity

**Figure 6:**
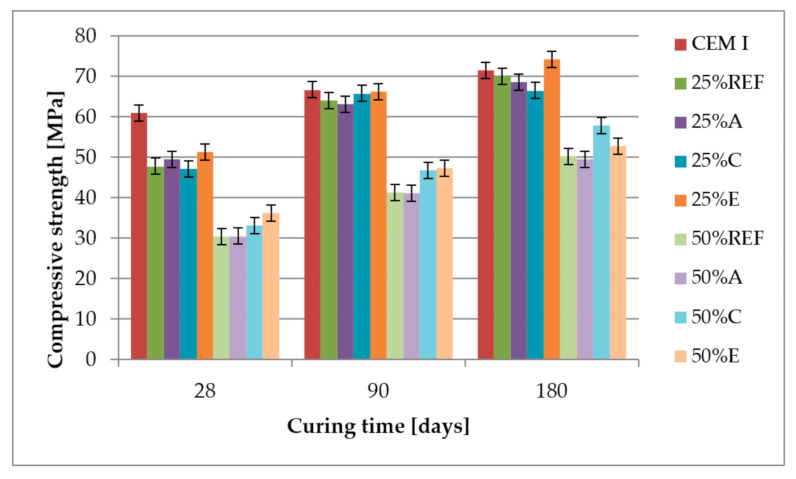
Compressive strength of mortars.

**Figure 7:**
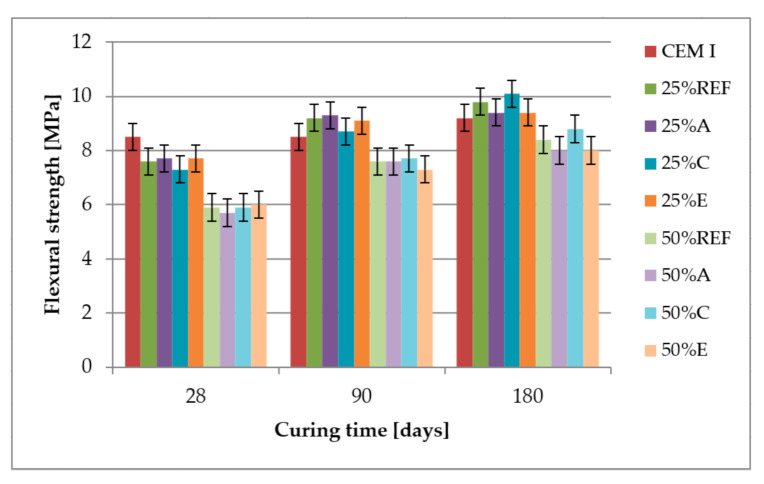
Flexural strength of mortars.

Figure 6 and Figure 7 present the results of compressive and flexural strength tests performed on mortars with the composition given in Table 5 after 28, 90 and 180 days of curing.

**Figure 8:**
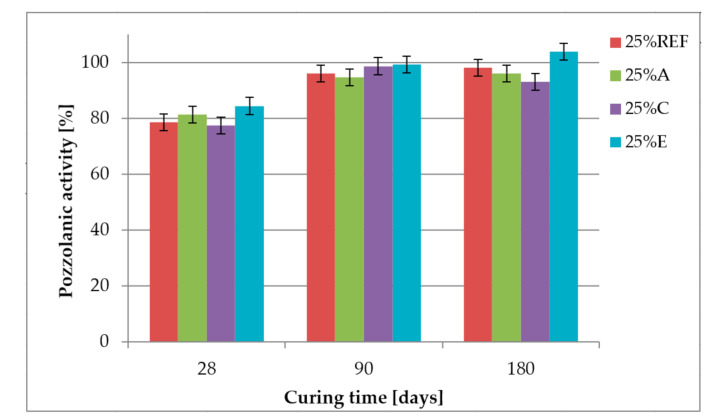
Activity index of fly ash.

The compressive and flexural strength of mortars containing 25% and 50% of fly ash after 28 days of curing was lower than the reference sample without fly ash. After 90 days of curing, all samples containing 25% of fly ash almost reached the values of compressive strength obtained by the reference sample, which is also shown in Figure 8, where the activity index almost reaches the value of 100%. The flexural strength of mortars containing 25% of fly ash after 90 days of curing is from 2% to 9% higher than the reference values. After 180 days of curing, compressive and flexural strength increases for an additional 10%. Mortars containing 50% of fly ash did not reach the strength of the reference sample even after 180 days of curing.

Analysing the results of compressive and flexural strength, there was no influence between those parameters and the concentration of ammonia in fly ash. Although Fawzi and Kareem [48] have recorded that adding 1% of ammonia to the water decreases the compressive strength and increases the flexural strength of concrete, the concentration of ammonia in their tests was much higher.

Figure 8 presents the activity index of fly ash, which is the relative compressive strength of mortars containing 25% of fly ash to the reference mortar without fly ash. The EN 450-1 standard [9] requires the activity index after 28 days to be not less than 75% and after 90 days not less than 85%. All samples, even those containing fly ash with 400 ppm of ammonia, fulfil these requirements. The influence of ammonia on the activity index was not observed compared to the reference fly ash without ammonia.

### 3.5. Emission of Ammonia from Mortars

**Figure 9:**
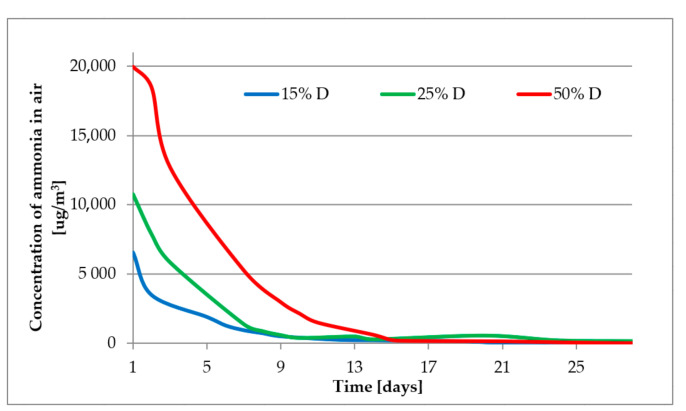
Emission of ammonia from mortars—sample D.

**Figure 10:**
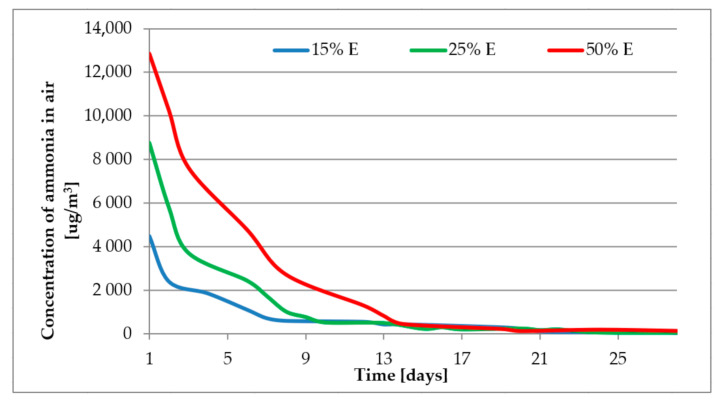
Emission of ammonia from mortars—sample E.

Figure 9 and Figure 10 present the results of emission of ammonia from mortars containing different amounts of fly ash, D and E, respectively. The composition of the tested mortars is given in Table 6.

The initial emissions from the samples of mortars containing 50% of fly ash reaches 21,000 and 13,000 µg/m^3^ for fly ash samples D and E, respectively. The concentration of ammonia in air falls over time and after about two weeks all registered values are at a level of about 300 µg/m^3^. After 28 days, when most of the concretes reach their declared parameters, the emission of ammonia from the tested mortars was negligible, even for those containing 50% b.m. of fly ash with 400 ppm of ammonia. Comparison of received levels of emission with literature data [17,22,30,49] is rather difficult due to different compositions of tested mortars/concretes, different initial amount of ammonia in composites and also different ways of measure the emission of ammonia. In our work we have tested the emission according to European standards but other researchers have performed their tests mostly according to their own procedures. The shape of our ammonia emission curves are similar to those of Shou et. al. [17] who observed that there is very high emission of ammonia during first days and a few days later the emissions rapidly fall. The emission of ammonia might be also related to the humidity. According to Figure 4 the highest emission are registered when the relative humidity is close to 90% and when humidity falls under 70% also the emission falls. This theory however needs to be proven in further experiments.

The requirements of the US Department of Health and Human Services [22,23] (less than 1 mg/m^3^) are fulfilled after 7 days for samples with 15% and 25% of fly ash, and after about 13 days for samples with 50% of fly ash. When the concentration is above 5 ppm an ammonia odour can be detected [26], which for samples 50% D and 50% E means a period of about one week. The most restrictive requirement stated in Polish national law [28] (300 or 100 µg/m^3^) might be fulfilled after 14–21 days.

## 4. Conclusions

After analysing the results of the performed tests, the following conclusions can be drawn:

- no correlation between the ammonia content and loss on ignition and the fineness of fly ash was found
- no significant effect of ammonia on mortar density and consistency was found
- no significant effect of ammonia on compressive strength and pozzolanic activity was found
- no significant influence of ammonia on water requirement was found
- intense release of ammonia gas was observed when the ash came into contact with a strongly alkaline cement grout, which might also cause the observed increase of the air content in fresh mortar
- ammonia in fly ash increases the initial and final setting times of the grouts within the limits permitted by the PN-EN 450-1 standard [1]. It was estimated, however, that ash containing more than 600 ppm of ammonia might extend the initial setting time not fulfilling the requirements of the standard
- ammonia present in the tested fly ash significantly affects the emission of this gas from mortars. It was observed that the amount of ammonia emission increases with the increase of ammonia concentration in the ash and the amount of added ash to the mortar. After 28 days, however, when the cement composites reach their declared parameters, the emission of ammonia is negligible

In answer to the question posed at the beginning of this paper, namely is siliceous fly ash containing up to 400 ppm of ammonia still a useful and safe material for concrete production, all tested fly ashes fulfilled the crucial requirements of the EN 450-1 standard [9], which means that they might be used as a concrete additive. After a period of 28 days, the emission of ammonia is very low and it is safe to use those composites. Special care, however, needs to be taken during the process of mixing and casting, and the initial days of curing the cement composites with ammonia fly ashes due to the emissions of harmful ammonia.

## Figures and Tables

**Table 1:** Ammonia content in the tested fly ash (A–E—samples from denitrification process).

Sample	Concentration in Water Solution (mg/L)	Concentration in Fly Ash (mg/kg)
REF	0.9	9
A	12.2	122
B	18.3	183
C	16.1	161
D	42.8	428
E	37.5	375

**Table 2:** Main properties of cement used for tests.

Property	Value
compressive strength (MPa)	
- 2 days	32.2
- 28 days	59.0
flexural strength (MPa)	
- 2 days	5.7
- 28 days	9.6
setting time (min)	170
end of setting time (min)	225
soundness expansion (mm)	0
loss on ignition (%)	3.30
insoluble residue (%)	0.90
specific surface area ^1^ (cm^2^/g)	3620
density (g/cm^3^)	3.10

^1^ Determined by the Blaine method.

**Table 3:** Composition of cement used for tests.

**Constituent**	SO_3_	Cl	SiO_2_	Al_2_O_3_	Fe_2_O_3_	CaO	MgO	K_2_O	Na_2_O	Na_2_O_eq_	C_3_A
**Content (%)**	2.82	0.052	19.96	5.00	2.47	64.37	1.27	0.70	0.13	0.59	9.07

**Table 4:** Composition of tested grouts.

Sample	Cement (g)	Fly Ash		Water (g)	Standard Consistency (%)	Standard Consistency (mm)
		(g)	Type			
CEM I	500	0	/–	128	25.5	5
25% REF	375	125	/Ref	127	25.5	4
25% A	375	125	/A	131	26.0	7
25% B	375	125	/B	131	26.0	6
25% C	375	125	/C	125	25.0	8
25% D	375	125	/D	126	25.0	5
25% E	375	125	/E	128	25.5	8

**Table 5:** Composition of tested mortars.

Sample	Cement (g)	Fly Ash (g)/Type		Water (g)	Sand (g)
CEM I	450.0	0.0	/–	225	1350
25% REF	337.5	112.5	/Ref		
25% A	337.5	112.5	/A		
25% C	337.5	112.5	/C		
25% E	337.5	112.5	/E		
50% REF	225.0	225.0	/Ref		
50% A	225.0	225.0	/A		
50% C	225.0	225.0	/C		
50% E	225.0	225.0	/E		

**Table 6:** Composition of mortars for the emission tests.

Sample	Cement (g)	Fly Ash (g)/Type		Water (g)	Sand (g)
15% D	382.5	67.5	/D	225	1.350
25% D	337.5	112.5	/D	225	1.350
50% D	225.0	225.0	/D	225	1.350
15% E	382.5	67.5	/E	225	1.350
25% E	337.5	112.5	/E	225	1.350
50% E	225.0	225.0	/E	225	1.350

**Table 7:** Physical characteristics of fly ashes.

Sample	Loss on Ignition (%)	Fineness (%)	Water Demand (%)
REF	1.43	34.2	99
A	4.02	36.2	101
B	4.47	32.2	101
C	2.43	29.6	100
D	3.36	26.4	100
E	3.47	25.8	100

**Table 8:** Properties of fresh mortar.

Sample	Density (kg/m^3^)	Air Content (%)	Consistency (mm)
100% CEM I	2.190	5.0	171
25% Ref	2.210	2.5	197
25% A	2.190	3.0	176
25% C	2.200	3.5	209
25% E	2.190	4.0	197
50% Ref	2.180	2.5	221
50% A	2.160	3.5	171
50% C	2.190	3.0	215
50% E	2.130	6.0	213

**Table 9:** Setting time of grouts.

Sample	Initial Setting Time		Final Setting Time	
	(min)	(%)	(min)	(%)
CEM I	165	100	225	100
25% Ref	185	112	320	142
25% A	215	130	345	153
25% B	230	139	355	158
25% C	230	139	345	153
25% D	305	185	415	184
25% E	255	155	365	162

## Data Availability

Data sharing not applicable. No new data were created or analyzed in this study.
